# Low-bias phosphopeptide enrichment from scarce samples using plastic antibodies

**DOI:** 10.1038/srep11438

**Published:** 2015-07-01

**Authors:** Jing Chen, Sudhirkumar Shinde, Markus-Hermann Koch, Martin Eisenacher, Sara Galozzi, Thilo Lerari, Katalin Barkovits, Prabal Subedi, Rejko Krüger, Katja Kuhlmann, Börje Sellergren, Stefan Helling, Katrin Marcus

**Affiliations:** 1 Medizinisches Proteom-Center, Ruhr-University Bochum, Universitätsstr. 150, 44801 Bochum, Germany; 2 Department of Biomedical Sciences, Faculty of Health and Society, Malmö University, SE 205 06 Malmö, Sweden; 3 Functional Neurogenomics Laboratory, Hertie-Institute for Clinical Brain Research, Eberhard Karls Universität Tuebingen and DZNE Tuebingen, Hoppe-Seyler-Str.3, 72076 Tuebingen, Germany; 4 Clinical and Experimental Neuroscience, Luxembourg Centre for Systems Biomedicine, University of Luxembourg, University of Luxembourg, 7, avenue des Hauts-Fourneaux, L-4362 Esch-sur-Alzette.

## Abstract

Phosphospecific enrichment techniques and mass spectrometry (MS) are essential tools for comprehending the cellular phosphoproteome. Here, we report a fast and simple approach for low sequence-bias phosphoserine (pS) peptide capture and enrichment that is compatible with low biological or clinical sample input. The approach exploits molecularly imprinted polymers (MIPs, “plastic antibodies”) featuring tight neutral binding sites for pS or pY that are capable of cross-reacting with phosphopeptides of protein proteolytic digests. The versatility of the resulting method was demonstrated with small samples of whole-cell lysate from human embryonic kidney (HEK) 293T cells, human neuroblastoma SH-SY5Y cells, mouse brain or human cerebrospinal fluid (CSF). Following pre-fractionation of trypsinized proteins by strong cation exchange (SCX) chromatography, pS-MIP enrichment led to the identification of 924 phosphopeptides in the HEK 293T whole-cell lysate, exceeding the number identified by TiO_2_-based enrichment (230). Moreover, the phosphopeptides were extracted with low sequence bias and showed no evidence for the characteristic preference of TiO_2_ for acidic amino acids (aspartic and glutamic acid). Applying the method to human CSF led to the discovery of 47 phosphopeptides belonging to 24 proteins and revealed three previously unknown phosphorylation sites.

Phosphorylation of proteins on the amino acids serine, threonine and tyrosine represents a post-translational modification (PTM) that defines protein function and the roadmap for intracellular signaling^1^,^2^. A comprehensive map of the cellular phosphoproteome is therefore of crucial importance for understanding cellular function as well as disease mechanisms (e.g., cancer or neurodegenerative diseases). Such endeavors rely on robust, highly sensitive methods capable of delivering quantitative and site-specific information on protein phosphorylation as a function of cellular status. Phospho-specific enrichment techniques and MS are essential tools for this purpose^3^,^4^,^5^. The large available repertoire of alternative phosphoselective enrichment techniques implies that current methods are far from perfect. Immuno-based methods are widely used for fractionation at the protein or peptide level. Currently, high-affinity antibodies for tyrosine phosphorylation are widely used, whereas the enrichment of phosphoserine/threonine-containing proteins has not been routinely possible because of the lower immunogenicity of the phosphoserine and phosphothreonine side chains^6^,^7^. Additionally, chemoaffinity protocols such as immobilized metal affinity chromatography (IMAC) or titanium dioxide (TiO_2_), although widely used in phosphoproteomics, lack site selectivity for phosphorylation at serine (pS), threonine (pT) or tyrosine (pY)^8^,^9^ and exhibit a sequence bias in favor of peptides rich in aspartic (D) and glutamic (E) acid. Moreover, these methods require access to a large amount, typically in the milligram range, of complex protein digest^5^,^10^,^11^.

Recently, we introduced a new approach for phosphopeptide and sulfopeptide enrichment featuring neutral, urea-based phosphate receptors prepared by molecular imprinting^12^,^13^. We demonstrated that the resulting molecularly imprinted polymers (MIPs) could in principle address the above deficiencies. In this context, a phosphotyrosine imprinted polymer (pY-MIP) was used to selectively enrich tyrosine-phosphorylated peptides spiked at low levels into proteolytic digests with only minor cross-reaction with pS peptides. However, this approach has yet to be used for biological samples or extended beyond pY recognition.

Using this versatile platform to engineer peptide receptors, we have now developed pS-MIP targeting serine phosphorylation and have challenged them, according to the protocol in Fig. 1, against state-of-the-art TiO_2_-based chemoaffinity methods for phosphopeptide enrichment. The orthogonality of the receptor was demonstrated in cross comparisons and experiments involving a standard peptide mixture, sequential elution and spiked mouse brain extracts. The versatility of the MIP receptors was then demonstrated for four different biological samples (trypsinized HEK 293T and SH-SY5Y cell lines, mouse brain and human cerebrospinal fluid (CSF)), paying special attention to the minimum required sample amounts, analytical throughput and amino acid sequence bias.

## Results

### Assessment of pS-MIP and pY-MIP for specific phosphopeptide enrichment

pS- and pY-MIPs were prepared using urea monomer 1 in a 2:1 stoichiometric ratio to the templates Fmoc-pSerOEt (2) or Fmoc-pTyrOEt (3), respectively, (Supplementary Figs 1, 2) in a manner similar to our previously reported procedure^12^,^13^. A nonimprinted polymer (NIP) was prepared identically to the imprinted polymers but without the template. These polymers exhibit minimal porosity in the dry state but significant swelling in acetonitrile (Supplementary Fig. 12). Imprinting effects were first assessed by chromatography using the crushed polymer monoliths as stationary phases. Thus, Fmoc amino acid derivatives were injected onto the columns in an acetonitrile-rich mobile phase acidified with trifluoroacetic acid (TFA) (Supplementary Fig. 3). The MIPs selectively retained pS and pY Fmoc derivates with a preference for the complementary amino acid side chains, whereas the nonimprinted polymer displayed weak retention of all analytes (Supplementary Fig. 4). This effect depended strongly on the aqueous content of the mobile phase. Whereas the pS-MIP showed optimum discrimination between Fmoc-pS and Fmoc-pY in a mobile phase containing 5% water, the pY-MIP operated best in more aqueous mobile phases. The latter result likely reflects a hydrophobic contribution to binding involving the aromatic tyrosine side chain.

Based on the mobile phase compositions that were identified as optimal for the amino acid solutes, we went on to probe for recognition of phosphorylated peptides using sequential elution, as shown in Fig. 2. The first goal was to use the pY- and pS-MIPs to fractionate a mixture of the mono- and multiply phosphorylated peptides listed (Supplementary Table 1) into three fractions: a pY peptide fraction, a pS peptide fraction and a fraction lacking phosphorylated peptides.

Given that the pY-MIP exhibited a slightly lower capacity than the pS-MIP, we decided to assess a sequential extraction protocol based on the pS- and pY-MIPs coupled in series. The resulting matrix-assisted laser desorption/ionization time-of-flight mass spectrometry (MALDI-TOF/TOF-MS) spectra of the flow-through and elution fractions are shown in Fig. 2. The orthogonality of the two solid phase extraction (SPE) steps is shown. Hence, the pS-MIP enriched 3 out of 4 of the serine-phosphorylated peptides, and the pY-MIP captured 3 out of the 4 tyrosine-phosphorylated peptides as well. Both MIPs exhibited a small degree of nonspecific binding to the other phosphopeptides, which was consistent with the template rebinding test (Supplementary Fig. 4). These experiments provided the first demonstration of site-selectivity of the polymers and encouraged us to study the phosphopeptides’ binding capabilities in more complex biological matrices.

To further elucidate the site selectivity of the pS-MIPs, we performed mouse brain protein lysate spike-in experiments (spiking level: 5 fmol to 2 pmol) with a phosphoserine peptide (SpS: AVP**S**PPPA**pS**PR) from the N-terminus of the mature serine protease HtrA2/Omi. To facilitate detection, the peptide was initially spiked at a high level into digested lysate (1.68 μg), and the samples were subjected to pS-MIP enrichment. The SPE flow-through and elution fractions were measured by MALDI-TOF/TOF-MS, resulting in the spectra shown in Fig. 3A and in Supplementary Figs 5 and 6. The absence of SpS in the flow-through fractions (Fig. 3) and the strong SpS signal in the otherwise clean elution fraction demonstrates the high SpS affinity of the pS-MIP. The elution fractions from lower-level spike-in solutions were subsequently measured using a more sensitive nanoLC-ESI-MS/MS system. The detection of spiked peptide at levels down to 5 fmol using this technique confirmed once again the effective capture of this peptide by the pS-MIP (Fig. 3B and Supplementary Figs 7–10).

### Enrichment of endogenous phosphopeptides from HEK 293T and SH-SY5Y samples

**Figure 1:**
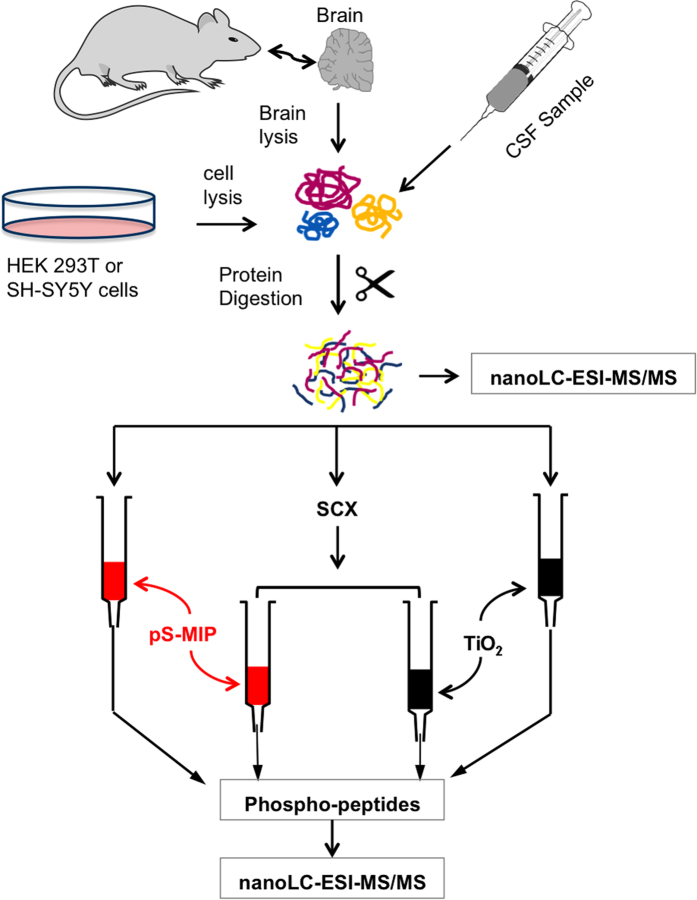
Work flow for phosphoproteomic analysis of harvested HEK 293T cells, mouse brain or CSF using SCX fractionation followed by pS-MIP or TiO_2_ enrichment. Samples (10 μg) of cell lysate or CSF tryptic digests were loaded before or after pre-fractionation with SCX onto pS-MIP or TiO_2_ columns for phosphospecific enrichment.

To assess the versatility and overall usefulness of the MIP-based phosphoenrichment approach, we performed an in-depth validation with respect to global phosphopeptide enrichment from biological samples according to the protocol outlined in Fig. 1. Cultured human embryonic kidney (HEK) 293T cells were first harvested from 10-cm dishes at 90% confluency at 37 °C; after cell lysis, the proteins were trypsinized into peptides. The tryptic fraction was then further fractionated using four alternative phospho-selective SPE protocols composed of pS-MIP, SCX and/or TiO_2_ phases. Pre-fractionation by strong cation exchange (SCX) chromatography was used to reduce sample complexity prior to more specific phosphopeptide enrichment (Supplementary Fig. 11). This step removes positively charged trypsinized peptides from the sample, while negatively charged peptides (e.g., phosphopeptides) are collected in the early eluting fractions. As shown in Fig. 4, a total of seven different fractions were analyzed, each with a minimum of three technical replicates. The fractions included the unfractionated lysate, the SCX-, TiO_2_- and pS-MIP-treated samples, and the samples obtained after fractionation by SCX followed by TiO_2_ or pS-MIP. The number of identified phosphorylated peptides was quantified using Proteome Discoverer V1.4; the analysis included a false-discovery rate approach and the phosphoRS 3.1 tool for assignment of phosphorylation sites. As shown in the bar graph in Fig. 4, 17 phosphopeptides were found in the unfractionated sample. A reduction of the complexity of the cell digest sample by SCX pre-fractionation alone roughly doubled the mean number of identified phosphopeptides compared with the untreated sample (from 17 to 39). After pS-MIP or TiO_2_ SPE on 10 μg of digested HEK 293T cell lysate, the phosphopeptide number increased to 237 or 330, respectively. However, this result contrasted with the result obtained by combining SCX- and pS-MIP-based fractionation. Using the combined technique, we observed 924 phosphopeptides, a drastic increase compared with pS-MIP or SCX alone. Technical replication (n = 3) of the analysis showed acceptable repeatability, with 610 phosphopeptides identified in all three measurements, with the ratio of serine-phosphorylated and threonine-phosphorylated peptides at about 9:1, indicating an imperfect selectivity of pS-MIP. We were able to reach a significantly higher number of identified phosphopeptides using pS-MIP compared to TiO_2_ enrichment with a modest but reproducible phosphopeptide recovery of up to 40%.

The yield of phosphopeptides using the combined SPE approach exceeded the yield obtained by TiO_2_-based SPE (with or without SCX pre-fractionation), regardless of whether low or high sample loads were used.

To assess the effectiveness of the pS-MIP approach in different cell types, we also analyzed a tryptic digest of the human neuroblastoma cell line SH-SY5Y. In this case, we were able to identify 1,403 phosphopeptides from 100 μg of digest via TiO_2_ SPE alone. By using pS-MIPs alone with 10 μg of peptides from the SH-SY5Y cell digest, we identified 648 phosphopeptides; by adding SCX pre-fractionation, we were able to enrich 1,271 phosphopeptides, a greater number than that obtained using HEK 293T cells. These data show that general phosphopeptide availability strongly depends on sample type.

The promising results using the plastic antibody-based enrichment approach were further confirmed by interpreting the data more stringently. The Venn diagram in Fig. 4 depicts the phosphopeptides identified using the different enrichment approaches while excluding unrepeatable phosphopeptide identifications. With respect to the five approaches, the results revealed capture phase- and sample load-dependent differences in phosphopeptide specificities. A total of 476 phosphopeptides were found exclusively by pS-MIP-based analyses (pS-MIP and SCX/pS-MIP), while the TiO_2_-based enrichments (TiO_2_, SCX/TiO_2_ and TiO_2_*) led to the identification of 274 TiO_2_-specific peptides. A GPMAW lite analysis was performed to determine the amino acid distributions in both groups of exclusively enriched phosphopeptides and in the untreated lysate sample (Fig. 5 and Supplementary Tables 2 and 3). As expected^14^, the peptides that were enriched using TiO_2_-based approaches displayed a sequence bias towards peptides containing the acidic amino acid residues aspartic acid (D) and glutamic acid (E). A more detailed analysis using the motif-x online tool^15^,^16^ revealed that the xxxSxxE and xxxSExx motifs were specific for TiO_2_. This bias was absent in the pS-MIP-enriched sample, with the notable exception of proline; motif-x revealed a preference for the xxxSPxx motif. Nevertheless, the phosphopeptides captured by pS-MIPs showed an amino acid distribution more closely reflecting that of the untreated sample.

Numerous peptides that were identified by multiple approaches were also detected, as well as 17 peptides that were present in all of the analyses. These 17 peptides were all phosphorylated at a serine and were likely present at a higher abundance, based on the numbers of spectra matched to each peptide (Supplementary Table 4).

### Enrichment of endogenous phosphopeptides from clinically relevant samples

Given the compatibility of the pS-MIP approach with low sample loads, we then focused our attention on the analysis of scarce biological and clinically relevant samples, using the SCX/pS-MIP enrichment approach to analyze 10 μg of SCX fractionated human CSF and mouse brain samples. In non-depleted CSF lysates, we identified 29 and 25 phosphopeptides from in-gel digested and in-solution digested samples, respectively. We also identified 18 phosphopeptides from depleted CSF (14 highly abundant proteins were depleted). Collectively, 47 phosphopeptides were detected from 24 proteins (Table 1), among which we report for the first time 8 phosphoproteins with a total of 19 phosphopeptides in human CSF. The 16 overlapping phosphoproteins confirmed former results published by Bahl *et al.* obtained by TiO_2_-based phosphopeptide enrichment of 200 μg filtered CSF protein digest^17^. In the mouse brain samples, a total of 673 phosphopeptides were identified, underlining the versatility of the SCX/pS-MIP approach for the analysis of complex biological and clinical samples.

## Discussion

The above results demonstrate the significant potential of MIPs as plastic antibody complements to established chemoaffinity-based (TiO_2_) or bioaffinity-based (antibodies) phosphopeptide capture and enrichment techniques.

We began by demonstrating the orthogonality of pS-MIPs and pY-MIPs for the amino acid side chain-selective fractionation of phosphorylated peptides. Focusing on the pS-MIP, we then investigated the sensitivity of the method for the detection of a proteotypic peptide from the serine protease HtrA2/Omi, a potential biomarker for Parkinson’s disease. The phosphoserine peptide could be detected at spiking levels as low as 5 fmol, highlighting the potential of the method for detecting low-abundance phosphopeptides in complex mammalian samples.

Focusing on endogenous peptides, analysis of HEK 293T cell lysate tryptic digests by SCX chromatography coupled with pS-MIP enrichment resulted in the identification of 924 phosphopeptides in only 10 μg of SCX fractionated peptide sample (starting material 40 μg trypsinized cell lysate). Larger numbers of phosphopeptides are typically found in large-scale phosphoproteomic studies employing multidimensional approaches (e.g., electrostatic repulsion-hydrophilic interaction chromatography (ERLIC), hydrophilic interaction liquid chromatography (HILIC), SCX/TiO_2_, and IMAC)^5^,^10^,^11^. However, these experiments have been performed on other cell types, starting with nearly 400-fold larger amounts of protein digest (4 to 15 mg). A study on human mesenchymal stem cell lysate (120 μg of tryptic digest) identified 350 phosphopeptides using the TiO_2_ technique, whereas sequential elution from IMAC captured 716 phosphopeptides^18^ Our results show that the total number of identified phosphopeptides varies significantly across sample types. For instance, TiO_2_ alone was able to enrich 711 phosphopeptides from 100 μg of HEK 293T cell lysate digest and 1,403 phosphopeptides from 100 μg of SH-SY5Y cell lysate digest. Similar number was reported by Montoya *et al.*^19^ with 1537 phosphopeptides detected by TiO_2_ after multiple enrichments using 1500 μg protein sample. The presented HEK 293T cell experiments showed that the SCX/pS-MIP enrichment approach resulted in a larger number of phosphopeptides (924) compared with the TiO_2_ approach (711) while using 10-fold less peptide sample. Moreover, the TiO_2_ approach showed a bias towards the acidic amino acids D and E, and motif-x analysis revealed the potential casein kinase motifs xxxSxxE and xxxSExx. Indeed, this preference for acidic residues has been confirmed by previous reports^14^. In contrast, the pS-MIP approach showed no obvious preference for acidic amino acid residues but instead showed a preference for a distinct hydrophobic SP-motif. Interestingly, this motif represents a major regulatory phosphorylation motif of Pro-directed protein kinases that encompasses numerous kinase classes, including CDKs, MAPKs, JNKs and GSK-3, that are involved in diverse cellular processes^20^.

We then demonstrated the versatility of the SCX/pS-MIP approach by applying it to scarce neurological research samples. By analyzing tryptic digests of CSF protein lysates, we were able to enrich 47 phosphopeptides (Table 1) belonging to 24 identified phosphoproteins. Previous research mapping the human CSF phosphoproteome using TiO_2_ capture resulted in the identification of 44 phosphoproteins^17^ however, our method complemented this list with 8 additional phosphoproteins. Three phosphorylation sites not annotated in UniProt, namely at Y-297 and S-298 in the Rho GTPase-activating protein 22 and at Y-84 in the immunoglobulin kappa chain C region are reported here for the first time. Identification of S-441 phosphorylation on amyloid-ß precursor protein A4 in CSF via SCX/pS-MIP enables further investigation of this marker in disease. Previously, we reported that amyloid-ß A4 is the binding partner of FE65, a cytosolic adapter protein whose interactome is of central interest in Alzheimer’s disease^21^. Moreover, phosphorylation of amyloid-ß A4 may play a critical pathological role. Finally, the compatibility of the SCX/pS-MIP approach with low sample inputs (a 60-μg sample of CSF protein material before trypsinization) makes it an attractive method for the study of proteins with relevance to neurodegenerative disease in CSF samples from diseased and control patients, which could in turn facilitate early-stage diagnosis. The analysis not only of CSF but also of brain samples could benefit from this approach. We identified more than 600 phosphopeptides in mouse brain samples, suggesting a use for the method in the analysis of post-mortem human brain samples.

## Conclusions

We have developed a new and highly versatile phosphopeptide enrichment technique that can be easily applied to scarce clinical samples. The SCX/pS-MIP method is unique with respect to its compatibility with low sample inputs, its programmable selectivity and its low sequence bias in combination with its robustness, speed and simplicity. Sample scarcity is common in neurological research dealing with human brain tissue and in the analysis of sub-fractions (e.g., neuromelanin granules^22^) or samples generated by laser microdissection of tissue^23^. Intriguingly, the plastic antibody was able to capture a large number of previously unknown phosphopeptides. We anticipate that this new approach will play a prominent role in future proteomics and diagnostics research.

## Methods

### Ethical Statement

The study of human cerebrospinal fluid was carried out in accordance with the approved guidelines with the request number 36/7/02 and 9/7/04. All experimental protocols were approved by the ethics committee at the Georg-August-University Goettingen, Germany. All participants provided written, fully informed consent to participate in this study.

### Sequential elution study using a standard peptide mixture

For material cleaning and conditioning, 20 mg of pS-MIP or pY-MIP were washed in a 1.5-ml reaction tube (Eppendorf) at room temperature with methanol (supplemented with 0.1% TFA) with medium-strength agitation in a thermomixer (Eppendorf) for 5 min, 2 h and overnight. Then, the sample tubes were centrifuged at 16,000 × g, and the supernatants were discarded after each washing step. The MIP conditioning was conducted twice for 10 min and followed by 2h of agitation with MeCN (supplemented with 0.1% TFA) in a thermomixer. For each washing and conditioning step, the slurry was centrifuged, and the supernatant was discarded.

**Figure 2:**
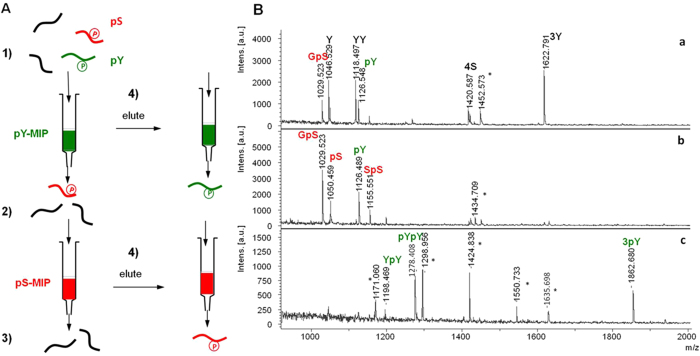
**A.** Sequential SPE workflow for fractionating a mixture of 12 phosphorylated and non-phosphorylated peptide standards using MALDI-TOF/TOF-MS for confirmation. **B**. MALDI-TOF/TOF-MS spectra of sequential SPE fractions. 1) The mixture was loaded on a pY-MIP column. 2) The flow-through fraction was collected and loaded on a pS-MIP column. 3) The flow-through fraction from the pS column was collected and measured (Fig. 2B, spectrum a). 4) Retained peptides from the MIP columns were eluted and measured (Fig. 2B, spectra b (pS-MIP) and c (pY-MIP)). Masses indicated with star (*) represent adducts or contaminants. 2S2pS could not be detected by MALDI-TOF/TOF-MS.

**Figure 3:**
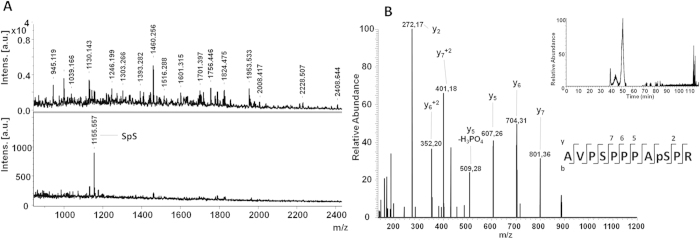
**A.** MALDI-TOF/TOF-MS identification of pS-MIP-treated SPE fractions of 0.5 pmol SpS peptide spiked into 1.68 μg of digested mouse brain lysate. **B**. NanoLC-ESI-MS/MS identification of pS-MIP-treated SPE fractions of 5fmol of SpS peptide spiked into 1.68 μg of digested mouse brain lysate. (**A**): Absence of SpS in the flow-through fraction (upper spectrum) and the strong SpS signal in the otherwise clean elution fraction (lower spectrum) demonstrates the high SpS affinity displayed by the pS-MIP. (**B**) Amino acid sequence showing typical prominent proline breakages allowing unequivocal SpS assignement. (Inset) Extracted ion chromatogram of spiked-in SpS.

For micro-column packing and solid phase extraction, a 2–200 μl pipette tip (Eppendorf) was filled at the bottom with a C8 membrane plug (3 M Empore^TM^ extraction disc, IVA Analysentechnik, Meerbusch, Germany). The conditioned pY- and pS-MIP material was vortexed in 500 μl MeCN (0.1% TFA), from which 15 μl of the slurry containing ca. 600 μg MIP was quickly pipetted into the pipette tip. After 3 min of sedimentation, the remaining solvent was removed with a 20 ml syringe (Terumo, Leuven, Belgien) equipped with a Chromabond^®^ adapter (Adapter PP-columns, Machery-Nagel, Düren, Germany). A mixture of 12 peptides (1 pmol each) (Supplementary Table 1) was loaded in loading solution (MeCN:H_2_O:TFA = 93:7:0.1) on the pY-MIP column. The flow-through was collected and loaded on the pS-MIP column. Bound peptides from the pY-MIP and pS-MIP columns were eluted as described below. The flow-through fraction and eluted fraction from the pY and pS columns were collected for MALDI-TOF/TOF-MS analysis (Fig. 2).

### Cell and mouse brain lysis

HEK 293T cells were cultured in 10-cm Petri dishes to 90% confluence before harvesting. Then, the cells were lysed on ice in 800 μl of lysis buffer 1, which was composed of 7 M urea (J.T.Baker), 30 mM tris(hydroxymethyl)aminomethane (Sigma-Aldrich), 2 M thiourea (Sigma-Aldrich), 0.1% Triton X-100 (Sigma-Aldrich), protease inhibitors (EDTA free cocktail tablet, Roche Diagnostics, added according to the manufacturer’s instructions) and phosphatase inhibitors [1 mM sodium orthovanadate (Sigma-Aldrich), 9.5 mM sodium fluoride (J.T.Baker) and 1 μM okadaic acid (Sigma-Aldrich)]. After homogenization using a EPPI-Pistille (Schuett biotec) for 1.5 ml reaction tubes, the cell lysate was sonicated 6 times for 10 s with 10-s intervals to avoid sample heating and then centrifuged at 16,000 × g for 15 min to remove the cell debris. The supernatant was collected, and the lysis process was repeated once with the pellet. The two supernatant fractions were subsequently pooled. Prior to in-gel digestion, the protein concentration was determined by Bradford protein assay (Bio-Rad, Germany) according to the manufacturer’s instructions, resulting in approximately 1-2 mg of extracted protein per cell dish. The mouse brain tissue was lysed using the same procedure in lysis solvent containing 7 M urea (J.T.Baker), 30 mM tris(hydroxymethyl)aminomethane (Sigma-Aldrich) and 2 M thiourea (Sigma-Aldrich). Lysis was conducted without detergents or phosphatase inhibitors for the spiking experiment and with phosphatase inhibitors for endogenous phosphopeptide analysis using the optimized SCX/pS-MIP combination.

### In-gel digestion

After cell or mouse brain lysis, a gel-based method was used to remove substances that interfere with MS, such as Triton X-100 (Sigma-Aldrich), from the sample. Then, 25 μg of cell or mouse brain lysate were mixed with 25% (v/v) lithium dodecyl sulfate (LDS, AppliChem) buffer [0.3 Mm LDS (AppliChem), 3.77 M glycerol, 563 mM TrizmaBase (Sigma-Aldrich), 423 mM TrizmaHCl (Sigma-Aldrich) and 1.6 mM EDTA (Merck)], heated at 95 °C for 5 min prior to loading and separated on acrylamide gels. The acrylamide gels were previously polymerized for 30 min in 1-mm-thick gel formats (XCell-SureLock^TM^ MiniCell system, Invitrogen). The gel solution contained 12.5% acrylamide (Serva) and 2.5 M bis(2-hydroxyethyl)amino–tris(hydroxymethyl)methane (Sigma-Aldrich), pH 6.8; polymerization was initiated after the addition of 1 μl of 40% (v/v) ammonium persulfate/ml of gel solution and 0.2 μl/ml tetramethylethylenediamine. The gel electrophoresis was performed at 50 V for 15 min in MOPS (AppliChem) buffer [50 mM 3-(N-morpholino)propanesulfonic acid (MOPS), 50 mM tris(hydroxymethyl)methane, 2% (v/v) SDS (AppliChem) and 1 mM EDMA]. After gel electrophoresis, the gel was stained with Coomassie Blue Imperial^TM^ Protein Stain (Thermo Scientific) for 10 min, and the background staining was reduced with water before the protein bands were excised, hashed and destained by alternating 10-min treatments with buffer A (10 mM ammoniumhydrogencarbonate, pH 8.3) and buffer B (buffer A:MeCN 50:50). Thereafter, the gel pieces were dried for 1 h in a rotational vacuum concentrator (RVC 2–25 CD, CHRIST), and a solution containing 12.5 μg trypsin (high-performance liquid chromatography (HPLC) purity > 90%, Serva) was added to the dried gel pieces (trypsin:protein = 1:20), followed by incubation at 37 °C for 16 h. The digestion was stopped and peptides extracted with 60 μl of extraction solution (50% MeCN, 0.1% TFA), followed by two sonication steps for 15 min in an ultrasonic bath and supernatant collection in glass vials. Afterwards, the pooled supernatants were dried in a rotational vacuum concentrator to remove the MeCN and re-dissolved in 0.1% TFA. Thereafter, the peptides were desalted using HyperSep C18 SpinTips (1–10 μl, Thermo Scientific) according to the manufacturer’s instructions, and the peptide concentrations were measured by amino acid analysis using AccQ Tag^TM^ derivatization of amino acids and subsequent separation and detection with an ACQUITY^TM^ UPLC system (Waters).

### CSF sample preparation

A CSF sample was prepared in one of three different ways prior to the application of SCX/pS-MIP: (i) the protein-containing sample (200 μg, 400 μl CSF) was tryptically digested^24^ in solution; (ii) the sample (200 μg) was depleted of the 14 most abundant proteins using SEP010 Seppro IgY14 Spin Columns (Sigma) followed by in-solution digestion; or (iii) the sample (150 μg) was separated (2 cm) by SDS-PAGE and sequestered by the prominent albumin band followed by in-gel digestion. After being desalted with HyperSep C18 SpinTips (Thermo Scientific), the peptide was pre-fractionated by SCX chromatography, yielding 30-35 μg peptide from the original 150–200 μg CSF protein sample.

### SCX chromatography

SCX chromatographic separations were performed on a Dionex U3000 HPLC System using a 5 μm SCX HPLC column (BioBasic, Thermo Scientific). Specifically, 160 μg of trypsin-digested HEK 293T lysate was lyophilized overnight and re-dissolved in 16 μl of SCX Solvent A (25% MeCN, 5 mM KH_2_PO_4_, pH 2.7), followed by loading onto the SCX Solvent A equilibrated column. After 5 min, the peptides were eluted with SCX Solvent B (25% MeCN, 5 mM KH_2_PO_4_, 500 mM KCl, pH 2.7) using a gradient from 0% to 50% B in 75 min followed by 50% to 100% B in 5 min and then maintained at 100% B for 10 min at a flow rate of 50 μl/min (Supplementary Fig. 11). To obtain a cationic compound-depleted fraction, the flow was collected from the 4^th^ to the 40^th^ min, lyophilized and desalted using HyperSep C18 SpinTips. Afterwards, the peptide concentration was determined by amino acid analysis before undergoing MIP- or TiO_2_-based phosphopeptide enrichment.

### Phosphopeptide enrichment with MIPs

Micro-columns were prepared as described above. Then, 10 μg of peptides from the HEK-293T cell digests, with or without SCX pre-fractionation, were loaded in the loading solvent (95% MeCN, 0.1% TFA) to the homemade SPE columns. After sedimentation for 3 min, the columns were placed in the pre-cut holes of 1.5 ml reaction tube caps and were centrifuged at 200 × g for 5 min in a tabletop centrifuge (Eppendorf 5415R) to spin the solution into the tubes. The loading procedure was repeated once, followed by two washing steps with the loading solution. Then, the peptides were eluted first with elution solution 1 (90% MeOH, 0.1% TFA) and then with elution solution 2 (50% MeOH, 0.1% TFA). The fractions were collected separately and dried in a rotational vacuum concentrator. The two eluates were pooled before they were completely dried. The dried fractions were finally re-dissolved in 0.1% TFA for MS analysis.

### Phosphopeptide enrichment with TiO_2_

For the TiO_2_ method, the setup and handling of the micro-columns were adapted from the MIP column treatment. All washing and eluting procedures were performed by centrifugation at 1,500 × g for 2 min. The peptides and TiO_2_ beads were applied at a ratio of 1:6 (e.g., 10 μg of peptides and 60 μg of TiO_2_) after 2 bead washing steps with TiO_2_ loading solution (80% acetonitrile, 5% TFA and 1 M glycolic acid). The peptide samples were loaded twice onto the columns by incubating for 3 min and subsequently removing the supernatant using a short centrifugation. Afterwards, the columns were washed twice with TiO_2_ loading solution, twice with TiO_2_ washing solution 1 (80% MeCN, 1% TFA), and then twice with TiO_2_ washing solution 2 (10% MeCN, 0.1% TFA). Three elution steps with increasing pH values were carried out using 50 μl of elution solvent 1 (250 mM ammoniumhydrogencarbonate alkalified with NH_4_OH to pH 9.1), elution solvent 2 (125 mM NH_4_HCO_3_ phosphoric acid alkalified with NH_4_OH to pH 10.5) and then elution solvent 3 (NH_4_OH in water, pH 11.3). After alkaline elution, the remaining peptides were eluted from the column membrane with 2 μl of 30% MeCN. All elution fractions were pooled and subsequently acidified with 15 μl 100% FA. Finally, the samples were desalted with C18 material and solubilized in 0.1% TFA for nanoLC-ESI-MS/MS experiments.

### MALDI-TOF/TOF-MS

MS measurement of the fractions collected during sequential elution and high-level spike-in experiments were performed using MALDI-TOF/TOF MS with a reflector TOF mass analyzer (Ultraflex II Bruker Daltonics). Fractions were collected in LC insert vials (HPLC CS-Chromatography Service GmbH) and dried in a rotational vacuum concentrator. After 10 min of drying, the two elution fractions were collected in one vial. The dried samples were re-dissolved in 5 μl of 0.1% TFA and mixed with 5 μl of MALDI matrix with additional ultrasonication for 10 min. Two microliters of this mixture were deposited in triplicate on the MALDI target plate (384 Anchor Chip target plate with Transponder Technology, Bruker Daltonik) and dried at room temperature. The MALDI matrix solution was prepared by dissolving 40 mg DHB (2,5-dihydroxybenzoic acid) in MeCN:Water (1:1 v/v, 1 ml) with 1% phosphoric acid and 0.1% TFA. The data were processed using the Flex Control software (Bruker Daltonics). Data collection, in terms of the laser and reflector voltage conditions and the number of scans, was performed identically for all samples unless otherwise noted. The spectra were collected by accumulating 400 laser shots under reflector mode and further analyzed with the Flexanalysis 3.0 software (Bruker Daltonics).

### Analysis of the SpS peptide spiked-in mouse brain digest

**Figure 4:**
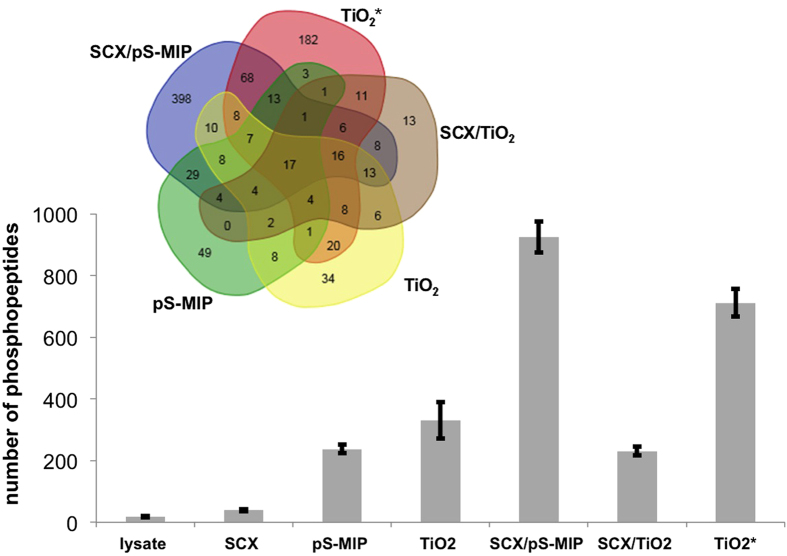
Mean number of phosphopeptides (bar graph) identified in 10 μg or 100 μg (TiO_2_*) of a tryptic digest of HEK 293T cell lysate by nanoLC-ESI-MS/MS analysis of a sample (lysate) before or after fractionation by SCX chromatography, pS-MIP SPE, TiO_2_ SPE or SCX followed by pS-MIP or TiO_2_ SPE as indicated. Combined SCX and pS-MIP fractionation resulted in the greatest phosphopeptide recovery. An overview of the phosphopeptides identified in all replicates (Venn diagram) revealed 17 common phosphopeptides that were detected independently of the fractionation technique and showed pronounced differences in selectivity among the different approaches.

**Figure 5:**
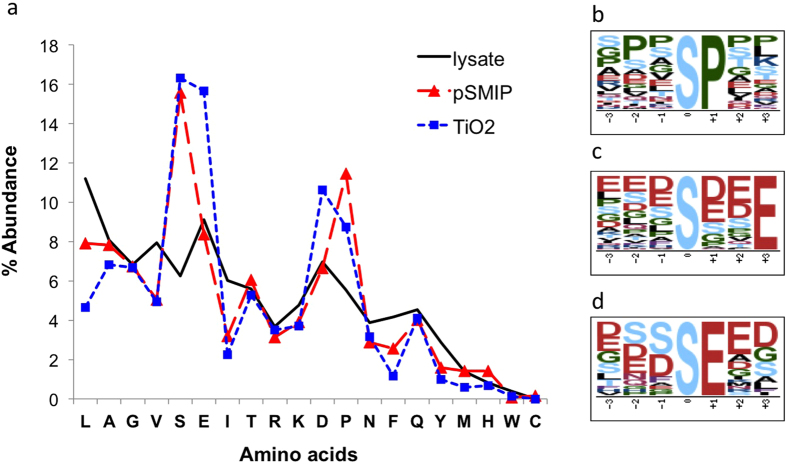
Characterization of phosphopeptides exclusively identified by the TiO_2_- or pS-MIP-based methods. (**a**) Amino acid distribution of the trypsinized peptides identified in the total lysate (black solid line), the 274 phosphopeptides exclusively identified by TiO_2_ (blue squares and dotted line) and the 476 phosphopeptides exclusively identified by pS-MIP-based analysis (red triangles and dashed line). (**b**) Common peptide motif of the phosphopeptides exclusively identified by pS-MIP. (**c**) and (**d**) Peptide motifs of the phosphopeptides exclusively identified by TiO_2_. Only peptides that were identified in all replicates were used for analysis. The graphical representations of the motifs in b, c and d were generated by motif-x for all 7-residue subsequences with a phosphorylated S at the center found in peptides identified by either enrichment method. Motif-x significance was set to 0.0005 and occurrences to 20. There were 38 threonine-phosphorylated peptides enriched by TiO_2_ approach, but due to the prerequisite of motif-x study that a minimum of 20 occurences could be considered as a motif, no xxxTxxx motif for phosphor-threonine peptides was identified.

The SpS peptide AVPSPPPApSPR was added to a complex mixture of 1.68 μg of trypsin-digested mouse brain lysate in concentrations ranging from 2 pmol to 5 fmol. MALDI-TOF/TOF MS analysis detected the SpS peptide ion that was strongly purified in the elution fractions down to 0.5 pmol, given by the ion of m/z = 1,155.556. The peptide identity was assigned by subsequent fragmentation experiments in the LIFT mode. Then, the precursor MS/MS spectrum of the 2 pmol eluate (Supplementary Fig. 7b) was compared with previous characterizations of the pure peptide (Supplementary Fig. 7a). The lower concentrations of 0.25 and 0.1 pmol were not detectable by MALDI-TOF/TOF MS. Therefore, to analyze the efficiency of peptide enrichment at concentrations below 0.5 pmol (100 fmol, 50 fmol, 10 fmol, 5 fmol), the elution fractions were measured on an Orbitrap Q-Exactive mass spectrometer using nanoLC-ESI-MS/MS (Supplementary Figs 8–10 and Fig. 4).

### NanoLC-ESI- MS/MS measurement

After cell lysis, SCX fractionation and pS-MIP or TiO_2_ SPE, the samples were measured by nanoLC-ESI-MS/MS. Nano HPLC analysis was performed on an UltiMate 3000 RSLC nano LC system (Dionex, Idstein, Germany) using the following solvent system: (A) 0.1% FA; (B) 84% ACN, 0.1% FA. The samples were first loaded on a trap column (Thermo, 100 μm × 2 cm, particle size 5 μm, pore size 100 Å, C18) with a flow rate of 30 μl/min of 0.1% TFA. After sample concentration and washing, the trap columns were serially connected with an analytical C18 column (Thermo, 75 μm × 50 cm, particle size 2 μm, pore size 100 Å), and the peptides were separated with a flow rate of 400 nl/min using a solvent gradient of 4% to 40% B for 95 min that remained for 5 min and was then returned to 4% B. After each sample measurement, 1 h of column washing was performed for equilibration. The HPLC system was online-coupled to the nano ESI source of a Q Exactive mass spectrometer (Thermo Fisher Scientific). In the ESI-MS/MS analysis, full MS spectra were scanned between 350 and 1,400 m/z with a resolution of 70,000 at 200 m/z to detect precursor ions (AGC target 3e6, 80 ms maximum injection time). The spray voltage was 1,600 V (+), and the capillary temperature was 250 °C. Lock mass polydimethylcyclosiloxane (m/z 445.120) was used for internal recalibration. The m/z values initiating MS/MS were set on a dynamic exclusion list for 30 s, and the top ten most intensive ions (charge state + 2, + 3, + 4) were selected for fragmentation experiments.

MS/MS fragments were generated by high-energy collision-induced dissociation (HCD) in which ion dissociation was performed at a normalized collision energy (NCE) of 27%, fixed first mass of 130.0 m/z and an isolation window of 2.2 m/z. The fragments were analyzed in an orbitrap analyzer with 35,000 resolution at 200 m/z (AGC 1e6, maximum injection time 120 ms).

### Data processing, database search and identification of phosphosites

For database searches, the raw files were analyzed with the ProteomDiscoverer 1.4 (Thermo Fisher Scientific) software using the Mascot V.2.3 search algorithm (Matrixscience, London UK) against the Uniprot/Swissprot database using human taxonomy (released 2013/10, 541,561 sequences in the whole database and 20,352 for human). The software-implemented Percolator was used to determine the false discovery rate (FDR), and the PhosphoRS 3.1 tool was applied to locate the phosphorylation sites. The following search parameters were used: peptide precursor mass ion selective range of 400–10,000 Da with mass tolerance of 5 ppm; fragment mass tolerance of 20 mmu; two allowed missed cleavages; two dynamic modifications at oxidation of methionine and phosphorylation at serine, threonine or tyrosine. The filter cut-off for the identified peptides was set at a targeted FDR of 1%.

### Analysis of phosphopeptide motifs

To characterize the phosphopeptides identified specifically by pS-MIP or TiO_2_ enrichment, we analyzed the frequency of each amino acid from each group and from the untreated lysate sample. The distribution calculation was performed using the free online bioinformatics tool GPMAW lite.

From a total of 952 identified phosphopeptides, 274 peptides were exclusively found when analyzing three different groups via TiO_2_ methods, while 476 unique peptides were found when analyzing the two groups of samples via the pS-MIP based method. To evaluate the physico-chemical properties of these exclusive sets and to distinguish them from each other, we applied the online tool motif-x. Motif-x identifies repetitive motifs in a foreground database (FG), which is derived from a group of peptides of interest (such as the TiO_2_-exclusive peptides or the pS-MIP-exclusive peptides) and evaluates their significance against a background database (BG). A significant motif will score a high frequency of matches in the FG and is specific in that it will simultaneously score a much lower frequency on the BG.

We conducted two motif-x runs for motifs with a length of 7 and requiring a phosphorylated S to be at the center. One run was for the TiO_2_-exclusive peptides and the other was for the pS-MIP-exclusive peptides. Such motifs are long enough to be meaningful but short enough to ensure that not too many phosphorylated serines are discarded because they are too close to the end of a peptide. For each of the two exclusive sets, we constructed an FG by extracting from all pertinent peptides all possible subsequences of length 7 containing a phosphorylated S at their centers. Analogously, we generated a BG database of all identified peptides. For the ‘occurrences’ parameter, we used with the motif-x default of 20. However, we chose a significance parameter of 5e-4 rather than the 1e-6 default, as the latter caused motif-x to yield no motifs. This relaxed stringency, as cautioned by motif-x, may lead to a higher order of false-positive motifs, as more background common-residue-position pairs are accepted into the set of reported motifs. In fact, Supplementary Table 2 reveals relatively low-fold increases in values. In this context the *fold increase*, as defined in literature^15^,^16^, is the quotient (number of FG matches/FG size)/(number of BG matches/BG size). In our case, the elements of the BG are tailored to a precise motif-length, and hence, there is less room for a given motif to fit into a BG element at random.

To independently verify the quality of the results, using a 5% level of significance and an FDR p-value adjustment, 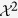-tests with Yates’ continuity correction were conducted to test each motif for dependency of FG/BG versus match/no-match, as derived from the values listed in Supplementary Table 2. These tests showed the specificity of all three motifs for their respective FGs. For greater detail, please see Supplementary Table 3.

The FG dataset for the pS-MIP-exclusive 7-residue subsequences centered on a phosphorylated S contained 219 elements. The FG for the TiO_2_-exclusive set contained 213 elements.

We also considered phosphorylated T and Y as center residues for the motifs. However, the numbers of these phosphorylated residues proved to be too small for reliable motif detection.

## Additional Information

**How to cite this article**: Chen, J. *et al.* Low-bias phosphopeptide enrichment from scarce samples using plastic antibodies. *Sci. Rep.* **5**, 11438; doi: 10.1038/srep11438 (2015).

## Supplementary Material

Supplementary Information

Supplementary Dataset 1

## Figures and Tables

**Table 1:** nanoLC-ESI-MS/MS-identified phosphoproteins present in a human CSF sample treated with SCX/pS-MIP and compared with the TiO_2_ approach^17^.

Number	Protein name	Protein Group Accessions	Found by TiO2
1	Golgi intergral membrane protein 4	O00461	no
2	Apolipoprotein L1	O14791	yes
3	Extracellular matrix protein 2	O94769	yes
4	Cystein C	P01034	yes
5	Ig kappa chain C region	P01834	no
6	Apolipoprotein E	P02649	yes
7	Serum Albumin	P02768	yes
8	Secretogranin-1	P05060	yes
9	Amyloid beta A4	P05067	yes
10	Osteopontin	P10451	yes
11	Chromogranin-A	P10645	yes
12	Clusterin	P10909	no
13	Coagulation Factor	P12259	no
14	Secretogranin-2	P13521	yes
15	Versican core protein	P13611	yes
16	Cadherin-2	P19022	yes
17	Inter-alpha-trypsin inhibitor heavy chain H2	P19823	yes
18	Actin,cytoplasmic	P60709	no
19	Nucleobindin-1	Q02818	no
20	SPARC-like protein 1	Q14515	yes
21	Rho GTPase-activating protein 22	Q7Z5H3	no
22	Golgi intergral membrane protein 1	Q8NBJ4	yes
23	Secretogranin-3	Q8WXD2	yes
24	Receptor-type tyrosine-protein phosphatase N2	Q92932	no
