# Supplementary material for: Low-bias phosphopeptide enrichment from scarce samples using plastic antibodies
Source: Sci Rep. 2015 Jul 1;5:11438. doi: 10.1038/srep11438 (PMC4486973; doi:10.1038/srep11438)
Supplement: Supplementary Information [file srep11438-s1.pdf]

Supplementary Information

Low-bias phosphopeptide enrichment from scarce samples using plastic antibodies

Jing Chen[a]*, Sudhirkumar Shinde[b]*, Markus-Hermann Koch[a], Martin Eisenacher[a], Sara Galozzi[a], Thilo Lerari[a],Katalin Barkovits[a], Prabal Subedi[a], Reijko Krüger[c, d], Katja Kuhlmann[a],Börje Sellergren[b]*, Stefan Helling[a]* and Katrin Marcus[a]*

Synthesis of molecularly imprinted polymers

N-(9-Fluorenylmethoxycabonyloxy) succinimide (Fmoc-OSu) and serine ethyl ester hydrochloride (Ser-OEt HCl) were obtained from Bachem; benzyl alcohol (99%) was obtained from Across Organics, and N,N-diisopropylethyl amine (DIEA), tert-butyl hydroperoxide (5.0-6.0 M solution in decane), and tetrazole solution were obtained from Fluka. The base 1,2,2,6,6-pentamethylpiperidine (PMP) was purchased from Fluka (Buchs, Switzerland). The initiator *N*,*N´*-Azo-bis-(2,4-dimethyl)valeronitrile (ABDV) was purchased from Wako Chemicals (Neuss, Germany). HPLC-grade methanol (MeOH) and MeCN were purchased from Acros (Geel, Belgium). Fmoc-Ser(PO_3H_2)-OH and Fmoc-Tyr(PO_3H_2)-OH were purchased from Bachem AG Switzerland and had a declared purity of >97%. Pentaerythritol triacrylate (PETA) was purchased from Sigma-Aldrich (Steinheim, Germany).

N-(9-Fluorenylmethyloxycarbonyl) serine ethyl ester (Fmoc-Ser-OEt)

Fmoc-OSu (5 g, 14.82 mmol) and serine ethyl ester hydrochloride (2.514 g, 14.822 mmol) were added to 20 ml of dry dichloromethane and stirred under nitrogen atmosphere. Afterwards, N,N-diisopropylethyl amine (2.579 g, 14.86 mmol) was added to the reaction mixture at 0°C and subsequently stirred at room temperature for 12 hours followed by washing steps with 1 M hydrochloric acid, saturated sodium bicarbonate, water and brine; the reaction was then dried over sodium sulfate. The solvent was evaporated, and the compound precipitated from n-hexane yielded 3.69 g of white product (68.6% yield). ^{1}H NMR (400 MHz $CDCl_3$): δ 1.21-1.31 (t, 3H), 3.94-3.99 (d, 2H), 4.21 (m, 3H), 4.43-4.44 (m, 3H), 5.76-5.78 (d, 1H), 7.30-7.78 (m, 8H, Aromatic). ^{13}C NMR (100 MHz $CDCl_3$): δ 14.28, 47.25, 56.22, 62.14, 63.52, 67.31, 120.12, 120.14, 125.20, 127.19, 127.22, 127.87, 141.41, 141.45, 143.77, 143.93, 156.38, 170.58. LCMS observed mass m/z: M+18 (355.14+18) 373.17.

Di-benzyl-N,N-diisopropylphosphoramidate

Di-benzyl-N,N-diisopropylphosphoramidate was synthesized according to the procedure reported by Perich *et al*.[1] A solution of benzyl alcohol (0.02 mol) and triethyl amine (0.04 mol) in absolute diethyl ether (20 ml) was added slowly to a solution of (diisopropylamino) dichlorophosphine (0.01 mol) in 10 ml of absolute diethyl ether while maintaining the reaction temperature at 0°C. After complete addition, the solution was stirred at room temperature for 3 hours. The reaction mixture was then consecutively washed with 5% sodium bicarbonate and brine. After drying with anhydrous sodium sulfate, the solution was filtered and evaporated under reduced pressure, yielding 3.12 g of colorless liquid (90% yield). The obtained crude product was used for the subsequent reaction without further purification. ^{1}H NMR (400 MHz $CDCl_3$): δ 1.20-1.22 (d, 12H), 3.66-3.75 (m, 2H), 4.67-4.89 (m, 4H), 7.24-7.38 (m, 10H). ^{13}C NMR (100 MHz $CDCl_3$): 24.60, 24.67, 43.00, 43.12, 65.25, 65.43, 126.95, 127.19, 128.20, 139.45, 139.53. ^{31}P NMR ($CDCl_3$): δ 148.55.

N-(9-fluorenylmethyloxycarbonyl)-O-phosphoserine ethyl ester (Fmoc-Ser(PO_3H_2)-OEt)

Di-benzyl-N,N-diisopropylphosphoramidate (1.22 g, 3.532 mmol) dissolved in 5 ml of anhydrous tetrahydrofuran was added to a solution of Fmoc-Ser-OEt (0.5 g, 1.40 mmol) and tetrazole (9.38 ml, (0.45 M in acetonitrile, 4.22 mmol) in 15 ml of anhydrous tetrahydrofuran under nitrogen. The resulting solution was stirred at room temperature for 12 hours. t-Butyl hydroperoxide (1 ml of a 5-6 M solution in decane, 5 mmol) was added to this mixture at 0°C, and it was stirred for another 2 hours at 0°C. The reaction was further stirred at room temperature for an additional hour. After aqueous workup, the residue was dried and dissolved in 30 ml ethanol. A 10% Pd (weight %) on carbon (0.5 g) catalyst was then added, and the mixture was stirred under a hydrogen atmosphere more than 12 hours. The catalyst was removed over Celite, and the residue was further purified by silica gel column chromatography, eluted with chloroform:methanol:acetic acid 98:2:1. The purification yielded 0.302 g of a white compound (49% yield). ^{1}H NMR (400 MHz DMSO): d6; 1.15-1.20 (t, 3H), 4.10-4.32 (m, 6H), 4.87-4.89 (m, 1H), 5.66 (s, 1H), 7.33-7.91 (m, 8H, aromatic). ^{13}C NMR (100 MHz, DMSO d6): 14.06, 46.58, 54.62, 60.97, 64.32, 66.02, 120.15, 125.32, 127.13, 127.69, 128.28. δ ^{31}P NMR (DMSO d6): δ 0.12 (s). δ ^{31}P NMR ($(CD3)_2CO+CD_3OD$): δ 0.66 (s). MALDI-TOF observed m/z, $M+K^{+}+H^{+}$, M+2Na+, $M+Na^{+}+K^{+}$, $M+3Na^{+}$: 475.01, 481.1, 497.2 and 503.4, respectively; DESI-MS, M, $M+H^{+}$, $M+2H^{+}$, $M+3H^{+}$: 435.2, 436.12, 437.1 and 438.12, respectively.

Polymer preparation

The pY-MIP and pS-MIP were prepared following a similar protocol to that previously published.[2] In brief, the pS-MIP was prepared in the following manner. The bis-pentamethylpiperidine salt of Fmoc-pSer-OEt (template) (0.5 mmol), urea monomer (1

mmol), acrylamide (1 mmol) and pentaerythritol triacrylate PETA (13.3 mmol) were dissolved in tetrahydrofuran THF (5.6 ml) and the initiator azobis(2,4-dimethyl)valeronitrile (ABDV) (1% w/w of total monomers) was added to the solution. The solution was then transferred to a glass ampoule at 0°C, and the solution was purged with dry nitrogen for 10 minutes. The tubes were then flame-sealed while kept on ice, and polymerization was initiated by placing the tubes in a temperature-controlled water bath pre-set at 50°C. After 24 hours, the tubes were broken and the polymers were lightly crushed. They were thereafter washed 3 times with MeOH and extracted in a soxhlet apparatus with 1:1 methanol: 0.1 N HCl for 24 hours. This step was followed by further crushing and sieving, and the fraction from 36-50 µm was used for packing HPLC columns. A non-imprinted polymer (NIP) was prepared in the same manner as described above, but with the omission of the template molecule from the pre-polymerization solution. The polymers were characterized by nitrogen sorption analysis, swelling ratio measurement and by scanning electron microscopy (Supplementary Fig. 12) as reported elsewhere[2-4].

HPLC Evaluation

The 36-50-µm particle size fraction was sedimented repeatedly in 80:20 (v:v) MeOH:water to remove fine particles, and the slurry was then packed into HPLC columns (30 x 4.6 mm) using the same mixture as the pushing solvent. Subsequent analyses of the polymers were performed using an Agilent HP1050 or HP1100 system equipped with a diode array-UV detector and a workstation. Analyte detection was performed at 260 nm with a flow rate of 0.5 ml/min washing solution after injecting 5 µl of analyte stock solutions. The retention factor (k) was calculated as $k = (t-t_0)/t_0$, where t is retention time of the analyte and t_0 = retention of the void marker (acetone). Prior to each run, the columns were washed with MeOH (supplemented with 0.1% TFA) for at least 2 hours and equilibrated for at least 20 minutes with the mobile phase.

References

1. Perich J.W., Alewood P.F., Johns R.B. Synthesis of casein-related peptides and phosphopeptides. VII. The efficient synthesis of ser(P)-containing peptides by the use of Boc-SerPO$_3$R$_2$)-OH derivatives. *Aust. J. Chem.* **44** (2), 233-252 (1991).
2. Shinde, S., Bunschoten, A., Kruijtzer, J.A.W., Liskamp, R.M.J. & Sellergren, B. Imprinted polymers displaying high affinity for sulfated protein fragments. *Angew. Chem. Int. Ed. Engl.* **51**, 8326-8329 (2012).
3. Helling, S. *et al.* Ultratrace enrichment of tyrosine phosphorylated peptides on an imprinted polymer. *Anal. Chem.* **83**, 1862-1865 (2011).

4. Emgenbroich, M. *et al.* A phosphotyrosine-imprinted polymer receptor for the recognition of tyrosine phosphorylated peptides. *Chemistry*. **14**, 9516-9529 (2008)

Supplementary Figures

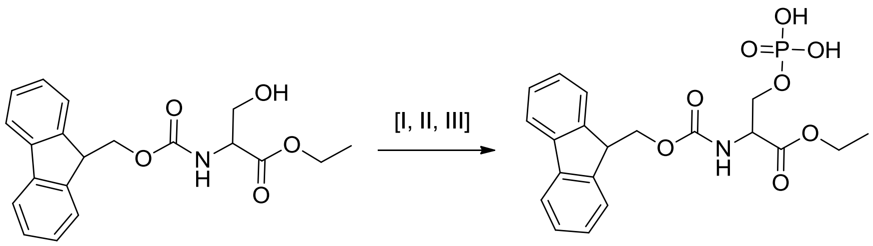

Supplementary Fig. 1 | Synthesis of the template Fmoc-pSer-OEt. [I] Di-benzyl-N,N-diethylphosphoramidate/tetrazole. [II] t-BuOOH, 0 °C. [III] H_2/ 10% Pd on charcoal.

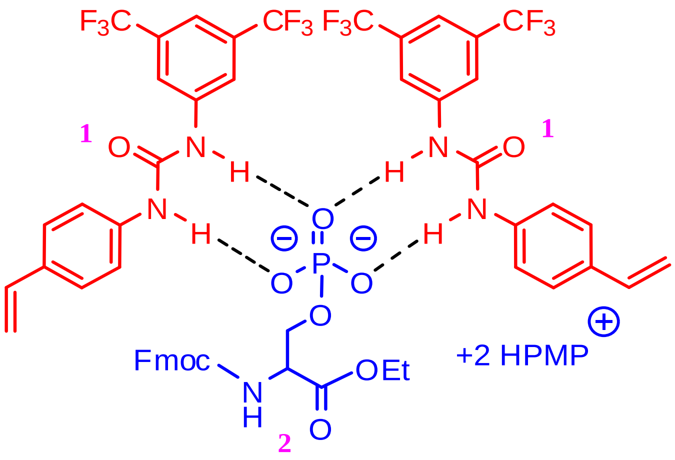

Supplementary Fig. 2 | Proposed prepolymerization complex of bis pentamethyl piperidinium of Fmoc-pSer-OEt (2) and urea monomer (1) in tetrahydrofuran.

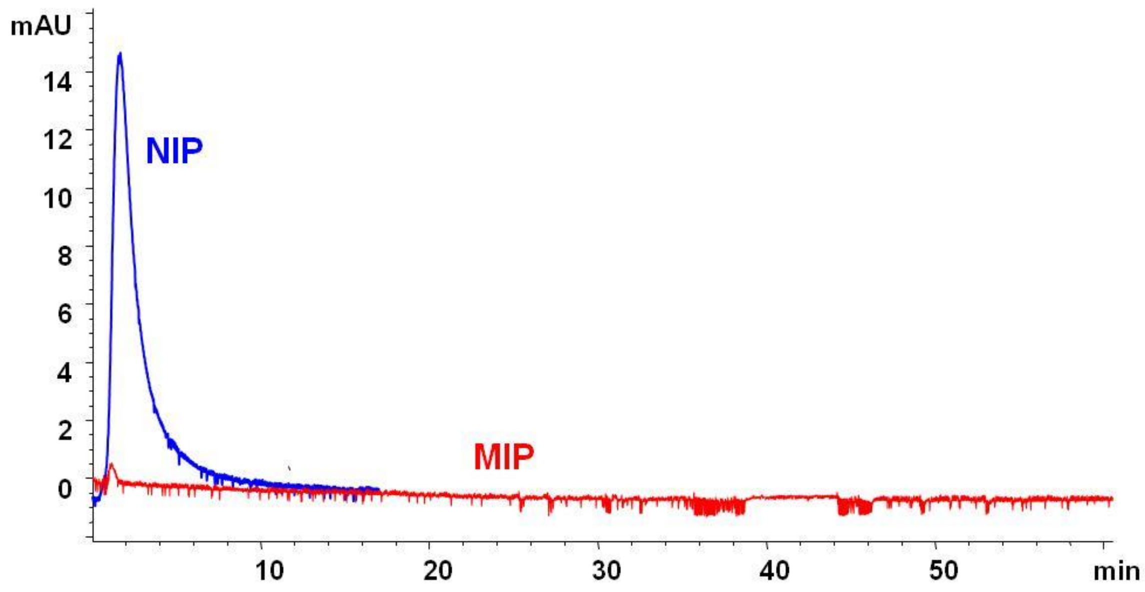

Supplementary Fig. 3 | HPLC binding test of the phosphoserine derivative Fmoc-pSer-OH on MIP and NIP polymers in acidic buffered mobile phase (MeCN:water:TFA=95:5:0.1). Injection volume: 5 µl; flow rate: 0.5 ml/min; DAD: 254 nm.

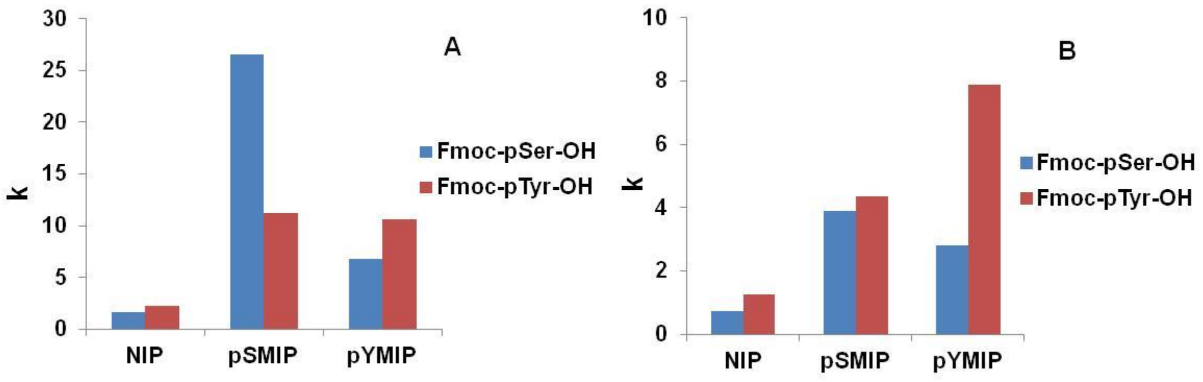

Supplementary Fig. 4: | Retention factor (k) of Fmoc-protected amino acids injected onto pS- and pY- MIP and NIP columns using different mobile phases. (A) (MeCN:water:TFA=95:5:0.1) (B) (MeCN:water:TFA = 50:50:0.1). Injection volume: 5 µl; flow rate: 0.5 ml/min; DAD: 254 nm.

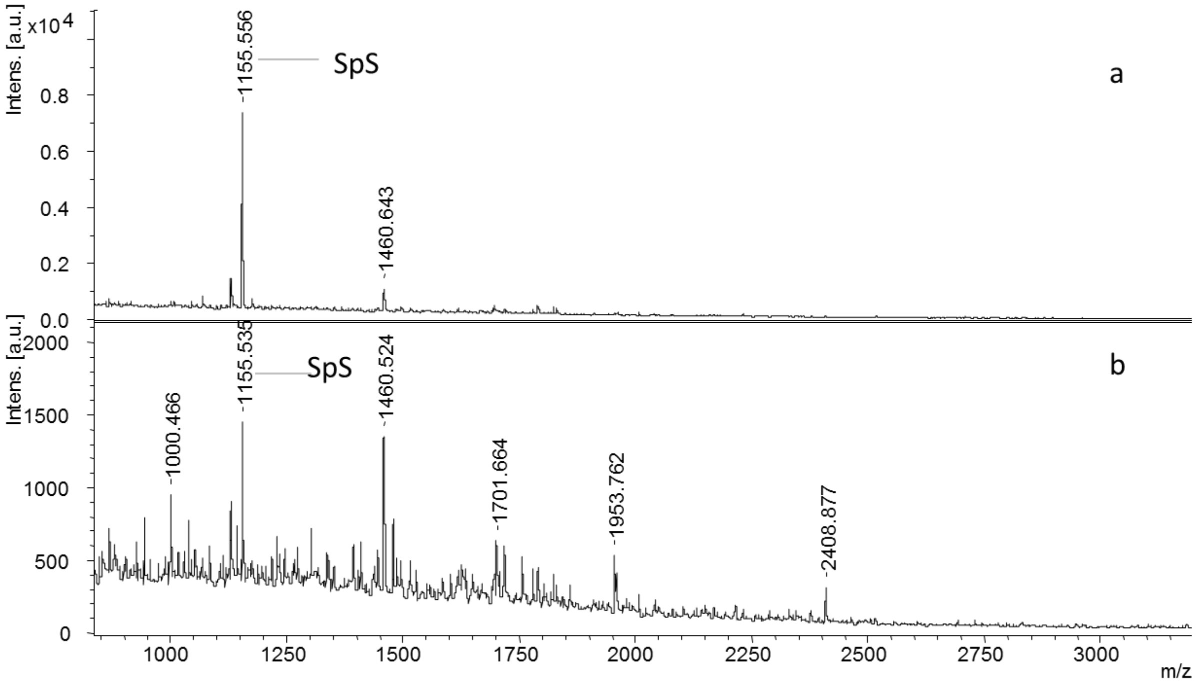

Supplementary Fig. 5 | MALDI-TOF/TOF-MS spectra of pS-MIP-treated SPE fractions of 2 pmol SpS peptide spiked into 1.68 µg digested mouse brain lysate. (a) Elution fraction; (b) flow-through fraction. The presence of SpS indicates overloading.

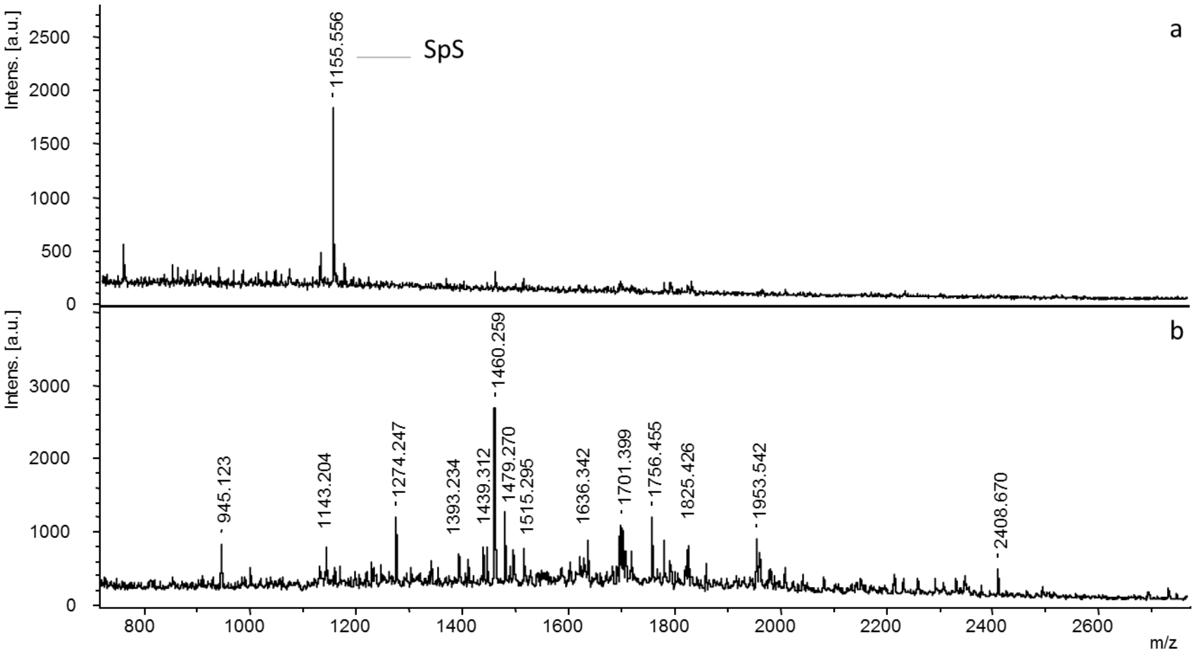

Supplementary Fig. 6 | MALDI-TOF/TOF-MS spectra of pS-MIP SPE fractions of 1 pmol SpS peptide spiked into 1.68 µg digested mouse brain lysate. (a) Elution fraction; (b) flow-through fraction.

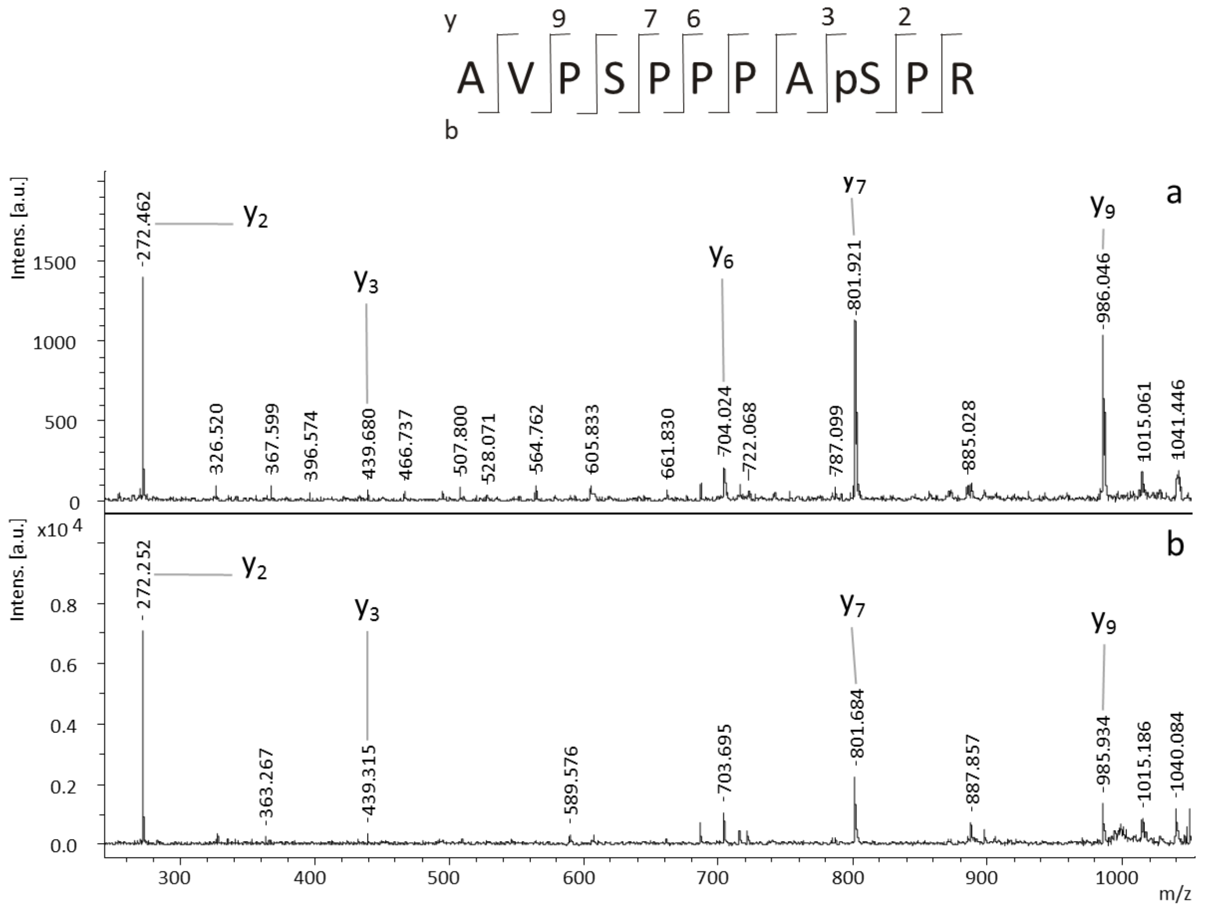

Supplementary Fig. 7 | MALDI-TOF/TOF-MS/MS spectra for confirming the identity of SpS m/z=1155.556, as assisted by theoretical fragment interpretation and by reference examinations using MS/MS fragmentation of the pure peptide. (a) MS/MS fragmentation of the standard peptide precursor ion. (b) MS/MS fragmentation of the precursor ion in the elution fraction of the 2 pmol spiking experiment.

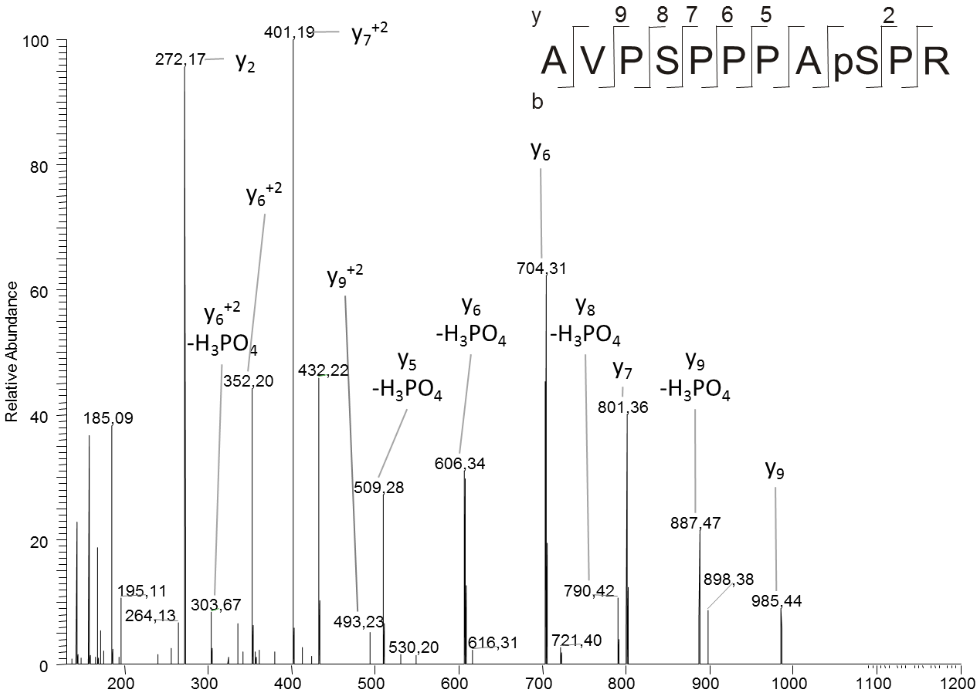

Supplementary Fig. 8 | NanoLC-ESI-MS/MS spectrum of the pS-MIP elution fraction after loading a sample with 100 fmol of SpS peptide spiked into 1.68 µg of mouse brain lysate. SpS:lysate = 6.8:10,000 (w/w).

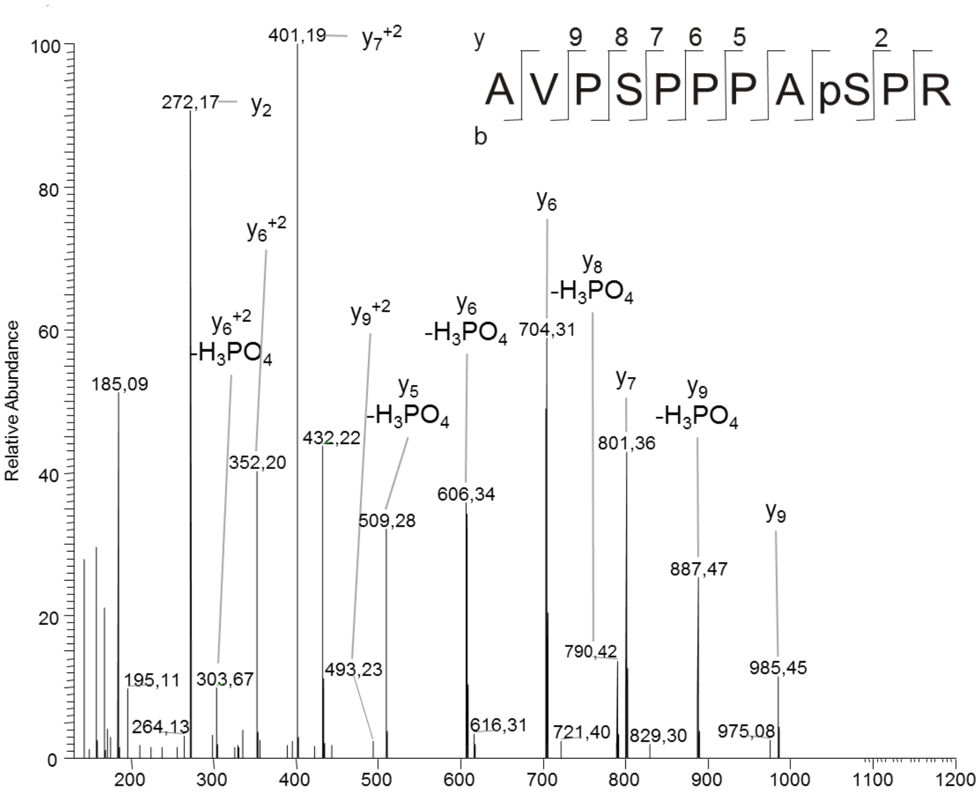

Supplementary Fig. 9 | NanoLC-ESI-MS/MS spectrum of the pS-MIP elution fraction after loading a sample with 50 fmol SpS peptide spiked into 1.68 µg mouse brain lysate. SpS:lysate = 3.4:10,000 (w/w).

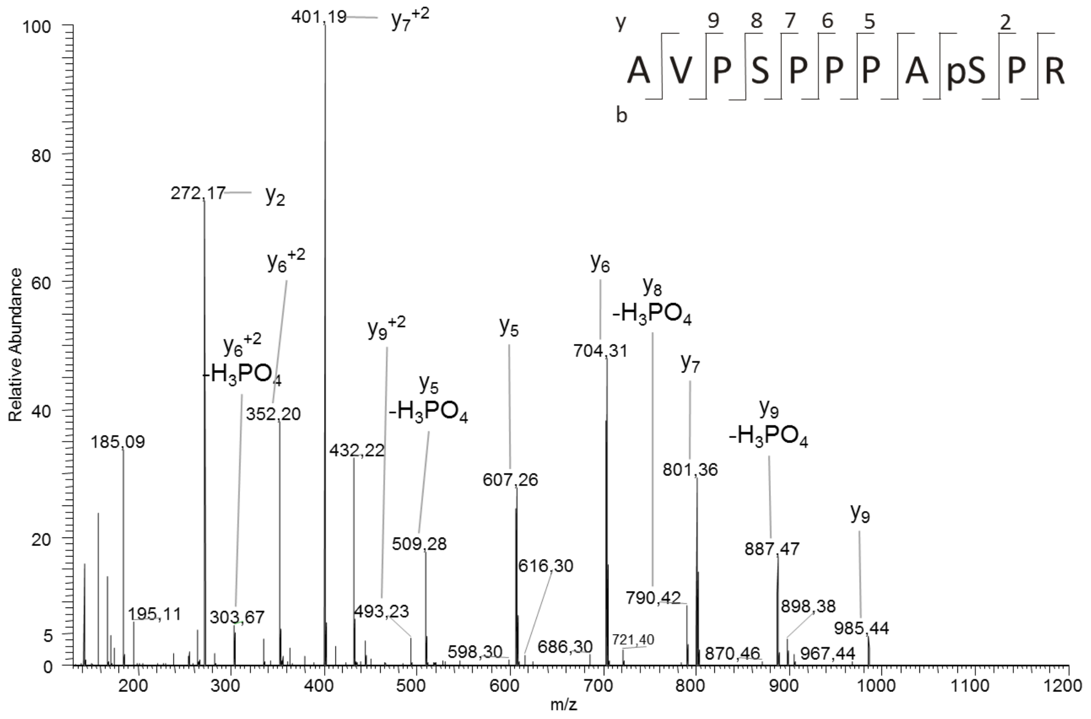

Supplementary Fig. 10 | NanoLC-ESI-MS/MS spectrum of the pS-MIP elution fraction after loading a sample with 10 fmol SpS peptide spiked into 1.68 µg mouse brain lysate. SpS:lysate = 6.8:100,000 (w/w).

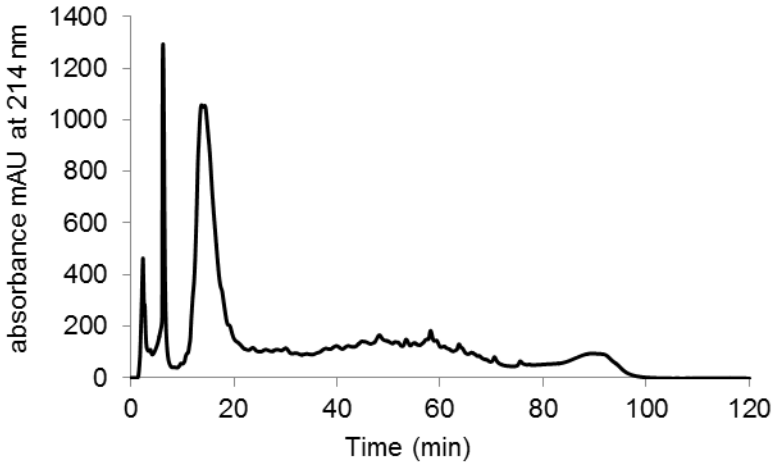

Supplementary Fig. 11 | Representative chromatogram after strong cation exchange (SCX) chromatography of tryptically digested HEK 293T cell lysates. The fraction eluting between 4 and 40 minutes was collected and used for subsequent pS-MIP SPE or for direct determination of the phosphopeptide content.

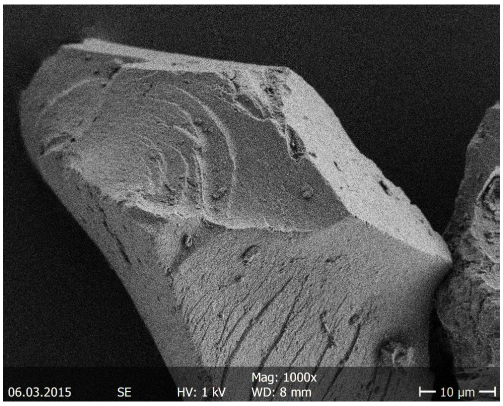

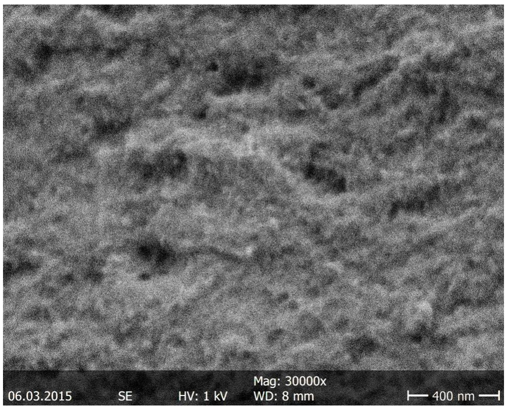

A. **B.**

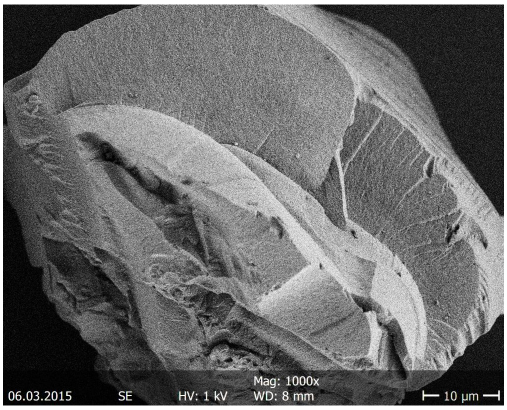

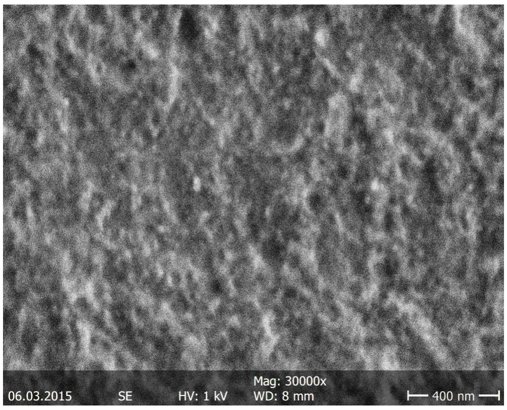

C. **D.**

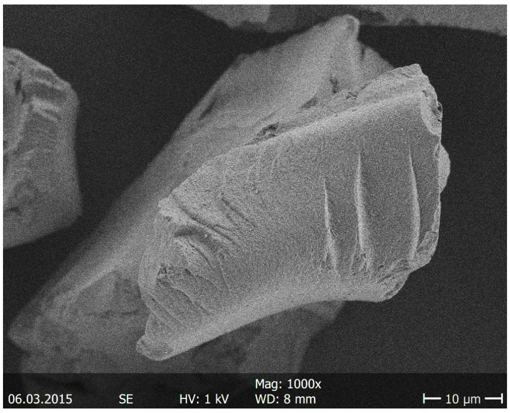

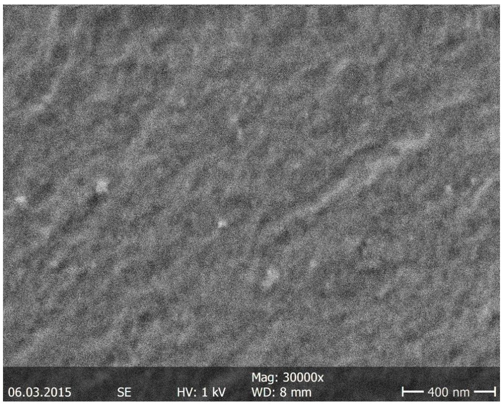

E. **F.**

Supplementary Fig. 12 | Scanning electron micrographs (SEM) of the pS-MIP (A, B), pY-MIP (C, D) and nonimprinted polymer NIP (E, F) at 1000x (A, C, E) and 30000x (B, D, F) magnification. BET specific surface areas of the polymers: pY-MIP: 23 m^2/g; pS-MIP: 11 m^2/g. Swelling ratio in MeCN/water: 95/5: pY-MIP: 1.72; pS-MIP: 1.33; NIP: 1.66.

Supplementary Table 1 | Tyrosine- and serine-containing model peptides used to probe phosphopeptide selectivity by SPE using pY-MIPs and/or pS-MIPs.

Peptide Sequence	Abbreviation	$[M+H]^{+1}$
VIL**GpS**PAHR	**GpS**	1029.5241
DRV**Y**IHPF	**Y**	1046.5418
DRV**pS**IHPF	**pS**	1050.4768
GADDS**YY**TAR	**YY**	1118.475
DRV**pY**IHPF	**pY**	1126.5081
AVP**S**PPPA**pS**PR	**SpS**	1155.5558
GADDS**YpY**TAR	**YpY**	1198.4412
GADDS**pYpY**TAR	**pYpY**	1278.4076
WWG**S**GP**S**G**S**GG**S**GGGK	**4S**	1420.624
WWG**S**GP**pS**G**S**GG**pS**GGGK	**2S2pS**	1580.5567
TRDI**Y**ETD**YY**RK	**3Y**	1622.7809
TRDI**pY**ETD**pYpY**RK	**3pY**	1862.6799

Supplementary Table 2 | Motif-X result for pS-MIP- and TiO_2-specific phosphopeptides using a motif length of 7, occurrences of 20, a S central residue and significance of 0.0005.

Dataset name	Motif	Motif score	FG matches	FG size at start of step	BG matches	BG size at start of step	Fold increase	% FG explained
pS-MIP	…SP..	4.92	114	219	196	519	1.38	52
TIO_2	…S..E	3.39	95	213	173	519	1.34	45
TIO_2	…SE..	3.72	22	118	29	346	2.22	10

Supplementary Table 3 | $\mathcal{X}^2$-testing ((FG/BG) versus (motif matches/motif does not match)) for all three motifs depicted in Supplementary Table 2. Counts were taken directly from Supplementary Table 2.

	Motif match	No motif match
FG (pS-MIP), …SP..	114	105
BG (all), …SP..	196	323
	p_{yates} = 0.04%	$p_{FDR\_adjusted}$ = 0.13%
FG (TIO_2), …S..E	95	118
BG (all). …S..E	173	346
	p_{yates} = 0.53%	$p_{FDR\_adjusted}$ = 0.53%
FG (TIO_2), …SE..	22	96
BG (all), …SE..	29	317
	p_{yates} = 0.36%	$p_{FDR\_adjusted}$ = 0.53%

Supplementary Table 4 | Repeatable nanoLC-ESI-MS/MS-identified phosphopeptides present in all pS-MIP and TiO_2 SPE experiments.

Sequence	Accession	PSM	Modification	PhosphoRS 3.1 Site probabilities*	Ion score§	$[M+H]^{+1}$
SSSPAPADIAQTVQEDLR	Q13283	69	S3(Phosp)	S(1): 0.4; S(2): 0.4; S(3): 99.2; T(12): 0.0	94	1964.89702
SASSDTSEELNSQDSPPK	O14745	47	S3(Phosp)	S(1): 0.4; S(3): 99.6; S(4): 0.0; T(6): 0.0; S(7): 0.0; S(12): 0.0; S(15): 0.0	113	1958.78569
EGEEPTVYSDEEEPKDESAR	O00264	45	S9(Phosp)	T(6): 0.0; Y(8): 0.0; S(9): 100.0; S(18): 0.0	49	2375.9395
HTGPNSPDTANDGFVR	P31943;P55795	44	S6(Phosp)	T(2): 0.0; S(6): 100.0; T(9): 0.0	81	1764.73381
FASDDEHDEHDENGATGPVK	P05455	39	S3(Phosp)	S(3): 100.0; T(16): 0.0	86	2249.8604
ESEDKPEIEDVGSDEEEEKK	P07900	36	S13(Phosp)	S(2): 0.0; S(13): 100.0	55	2400.98223
YGLQDSDEEEEEHPSK	P52948	32	S6(Phosp)	Y(1): 0.0; S(6): 100.0; S(15): 0.0	69	1971.74504
KPVTVSPTTPTSPTEGEAS	Q9Y6G9	32	S12(Phosp)	T(4): 0.0; S(6): 0.0; T(8): 0.0; T(9): 0.0; T(11): 0.0; S(12): 99.4; T(14): 0.6; S(19): 0.0	40	1965.90679
FEEESKEPVADEEEEDSDDDVEPITEFR	P54105	32	S17(Phosp)	S(5): 0.0; S(17): 100.0; T(25): 0.0	107	3394.35385
ESEDKPEIEDVGSDEEEEK	P07900	31	S13(Phosp)	S(2): 0.0; S(13): 100.0	48	2272.8897
NKPGPNIESGNEDDDASFK	O60841	30	S9(Phosp)	S(9): 100.0; S(17): 0.0	47	2113.87139
GNSRPGTPSAEGGSTSSTLR	P35269	25	S3(Phosp); T7(Phosp)	S(3): 100.0; T(7): 100.0; S(9): 0.0; S(14): 0.0; T(15): 0.0; S(16): 0.0; S(17): 0.0; T(18): 0.0	70	2078.85624
VFDDESDEKEDEEYADEK	O43719	23	S6(Phosp)	S(6): 100.0; Y(14): 0.0	91	2271.83306
FNDSEGDDTEETEDYR	Q9NYF8	21	S4(Phosp)	S(4): 100.0; T(9): 0.0; T(12): 0.0; Y(15): 0.0	112	2001.68633
SPVSTRPLPSASQK	Q8ND56	21	S1(Phosp)	S(1): 100.0; S(4): 0.0; T(5): 0.0; S(10): 0.0; S(12): 0.0	46	1534.76201
DHSPTPSVFNSDEER	Q6UN15	19	S3(Phosp)	S(3): 100.0; T(5): 0.0; S(7): 0.0; S(11): 0.0	61	1796.71306
SPVPSAFSDQSR	Q9UQ35	14	S1(Phosp)	S(1): 100.0; S(5): 0.0; S(8): 0.0; S(11): 0.0	50	1357.57854

PSM: peptide spectra match; *: probabilities of amino acid phosphorylation in %; §: maximal ion score of the Mascot search algorithm

Supplementary Table 5 | NanoLC-ESI-MS/MS-identified phosphopeptides present in a human CSF sample treated with SCX+pS-MIP, compared with the TiO_2 approach.[3]

Sequence	found by TiO_2	Protein name	Protein Group Accessions	Modifications
GREEHYEEEEEEEEDGAAVAEK	no	Golgi integral membrane protein 4	O00461	Y6(Phosp)
VTEPISAESGEQVER	yes	Apolipoprotein L1	O14791	T2(Phosp)
EALQSEEDEEVKEEDTEQKR	yes	Extracellular matrix protein 2	O94769	S5(Phosp)
EALQSEEDEEVKEEDTEQK	yes		O94769	S5(Phosp)
LVGGPMDASVEEEGVR	yes	Cystein C	P01034	M6(Oxidation); S9(Phosp)
VYACEVTHQGLSSPVTK	no	Ig kappa chain C region	P01834	Y2(Phosp)
AATVGSLAGQPLQER	no	Apolipoprotein E	P02649	T3(Phosp)
KVPQVSTPTLVEVSR	no	Serum Albumin	P02768	T7(Phosp)
ERADEPQWSLYPSDSQVSEEVK	no	Secretogranin-1	P05060	S13(Phosp)
GHPQEESEESNVSMASLGEK	yes		P05060	S7(Phosp); M14(Oxidation)
GEDSSEEKHLEEPGETQNAFLNER	yes		P05060	S5(Phosp)
GEDSSEEKHLEEPGETQNAFLNER	yes		P05060	S4(Phosp); S5(Phosp)
SAEFPDFYDSEEPVSTHQEAENEKDR	yes		P05060	S1(Phosp)
SQEESEEGEEDATSEVDKRR	yes		P05060	S1(Phosp)
SQEESEEGEEDATSEVDKR	yes		P05060	S5(Phosp)
VESLEQEAANER	yes	Amyloid beta A4	P05067	S3(Phosp)

KANDESNEHSDVIDSQELSK	no	Osteopontin	P10451	S6(Phosp)
ANDESNEHSDVIDSQELSK	yes		P10451	S5(Phosp); S14(Phosp)
ANDESNEHSDVIDSQELSK	yes		P10451	S5(Phosp)
ANDESNEHSDVIDSQELSKVSR	yes		P10451	S14(Phosp)
DSYETSQLDDQSAETHSHK	yes		P10451	S2(Phosp)
AIPVAQDLNAPSDWDSR	yes		P10451	S16(Phosp)
QNLLAPQNAVSSEETNDFKQETLPSK	yes		P10451	S11(Phosp)
QNLLAPQNAVSSEETNDFKQETLPSK	yes		P10451	S11(Phosp); S12(Phosp)
QNLLAPQNAVSSEETNDFK	yes		P10451	S12(Phosp)
EDSLEAGLPLQVR	no	Chromogranin-A	P10645	S3(Phosp)
VTTVASHTSDSDVPSGVTEVVVK	no	Clusterin	P10909	S11(Phosp)
EILSVDCSTNNPSQAK	no		P10909	S4(Phosp)
LEPEDEESDADYDYQNR	no	Coagulation Factor	P12259	S8(Phosp)
QQAHKEESSPDYNPYQGVSVPLQQK	no	Secretogranin-2	P13521	S8(Phosp)
EESSPDYNPYQGVSVPLQQK	no		P13521	S4(Phosp)
IESQTQEEVRDSKENIEK	yes		P13521	S12(Phosp)
QEVNPVRQEIESETTSEEQIQEEK	yes	Versican core protein	P13611	T15(Phosp)
ESAEVEEIVFPR	yes	Cadherin-2	P19022	S2(Phosp)
SLPGESEEMMEEVDQVTLYSYK	no	Inter-alpha-trypsin inhibitor heavy chain H2	P19823	S6(Phosp); M9(Oxidation); M10(Oxidation)
DLYANTVLSGGTTMYPGIADR	no	Actin, cytoplasmic	P60709	T13(Phosp)
AQRLSQETEALGR	no	Nucleobindin-1	Q02818	S5(Phosp)

EHANSKQEEDNTQSDDILEESDQPTQVSK	no	SPARC-like protein 1	Q14515	S14(Phosp)
HSASDDYFIPSQAFLEAER	no		Q14515	S11(Phosp)
MQEDEFDQGNQEQEDNSNAEMEEENASNVNK	yes		Q14515	M1(Oxidation); S17(Phosp); M21(Oxidation)
DQGNQEQDPNISNGEEEEEKEPGEVGTHNDNQER	yes		Q14515	S12(Phosp)
FLDEVQAYSNVNK	no	Rho GTPase-activating protein 22	Q7Z5H3	Y8(Phosp); S9(Phosp)
LRGEDDYNMDENEAESETDKQAALAGNDR	no	Golgi integral membrane protein 1	Q8NBJ4	M9(Oxidation); S16(Phosp)
ELSAERPLNEQIAEAEEDK	yes	Secretogranin-3	Q8WXD2	S3(Phosp)
ELSAERPLNEQIAEAEEDKIK	yes		Q8WXD2	S3(Phosp)
SEHPESSLSSEEETAGVENVK	no	Receptor-type tyrosine-protein phosphatase N2	Q92932	S10(Phosp)
